# Daily circadian misalignment impairs human cognitive performance task-dependently

**DOI:** 10.1038/s41598-018-20707-4

**Published:** 2018-02-14

**Authors:** Sarah L. Chellappa, Christopher J. Morris, Frank A. J. L. Scheer

**Affiliations:** 1 Medical Chronobiology Program, Division of Sleep and Circadian Disorders, Departments of Medicine and Neurology, Brigham and Women’s Hospital, Boston, MA 02115 United States; 2 Division of Sleep Medicine, Department of Medicine, Harvard Medical School, Boston, MA 02115 United States

## Abstract

Shift work increases the risk for human errors, such that drowsiness due to shift work has contributed to major industrial disasters, including Space Shuttle Challenger, Chernobyl and Alaska Oil Spill disasters, with extraordinary socio-economical costs. Overnight operations pose a challenge because our circadian biology inhibits cognitive performance at night. Yet how the circadian system modulates cognition over multiple days under realistic shift work conditions remains to be established. Importantly, because task-specific cognitive brain regions show different 24-h circadian dynamics, we hypothesize that circadian misalignment impacts cognition task-dependently. Using a biologically-driven paradigm mimicking night shift work, with a randomized, cross-over design, we show that misalignment between the circadian pacemaker and behavioral/environmental cycles increases cognitive vulnerability on sustained attention, cognitive throughput, information processing and visual-motor performance over multiple days, compared to circadian alignment (day shifts). Circadian misalignment effects are task-dependent: while they acutely impair sustained attention with recovery after 3-days, they progressively hinder daily learning. Individuals felt sleepier during circadian misalignment, but they did not rate their performance as worse. Furthermore, circadian misalignment effects on sustained attention depended on prior sleep history. Collectively, daily circadian misalignment may provide an important biological framework for developing countermeasures against adverse cognitive effects in shift workers.

## Introduction

We live in an “around-the-clock” 24/7 society, to the extent that ~10% of Americans work at night, on fixed, rotating, or irregular schedules^1,2^, and these numbers are only likely to increase. Epidemiological data suggest that up to a third of night shift workers meet the minimal diagnostic criteria for shift work disorder (SWD)^3^, defined by the International Classification of Sleep Disorders (ICSD-2) as a major circadian rhythm sleep disorder (CRSD)^4^. The consequences of working beyond our biological capabilities include symptoms ranging from wake-time sleepiness to disorders of sleep and cardiometabolic function^5–8^. Shift work increases the risk for human operational errors, such that drowsiness due to shift work has contributed to key industrial disasters, including Space Shuttle Challenger, Chernobyl and Alaska Oil Spill disasters^9^. Overnight operations challenge our circadian biology, which promotes daytime vigilance and nocturnal sleep^10^, thus resulting in a misalignment between the internal circadian (biological) rhythms and the required society demands for sleep/wake times^11^. Importantly, our endogenous circadian system inhibits cognitive performance in the biological nighttime hours^7^, although it remains to be fully established whether this increased cognitive vulnerability extends in a similar way across multiple cognitive domains or it is cognitive-domain specific^12^. Indeed, recent evidence suggests that fMRI-BOLD cognitive brain responses exhibit different 24-h circadian dynamics specific to a given task^13^, which highlights the task-dependent nature of the circadian deterioration in nighttime performance.

**Figure 1:**
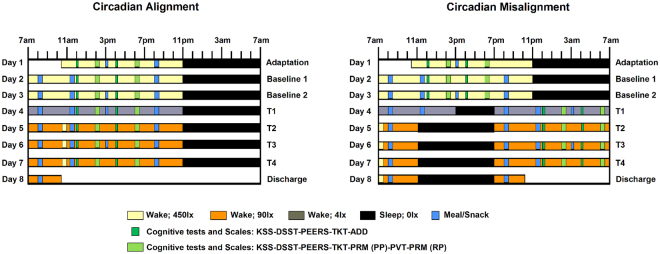
Within-subject, randomized, cross-over study design. Circadian alignment (**A**) and misalignment (**B**) protocols. For the former, scheduled sleep times were kept from 11PM to 7AM across all days, while for the latter these timings were inverted by 12 h after Baseline 2 (Day 3). T1-T4 corresponds to test days 1–4. During baseline days for both aligned and misaligned conditions, the Psychomotor Vigilance Task (PVT) and Probed Recall Memory (PRM) with Presentation phase (PP) and Recall phase (RP) were conducted at 2PM and 6PM, the Addition Task (ADD) at 12PM and 4PM, and the Unstable Tracking Task (TKT), Digit Symbol Substitution Task (DSST), Performance evaluation and effort scales (PEERS) and Karolinska Sleepiness Scale (KSS) at 12PM, 2PM, 4PM and 6PM. These timings were kept the same for the aligned protocol across all test days, while in the misaligned protocol they were inverted by 12 h for T1-T4.

Translating the putative role of circadian dynamics on human cognition from circadian protocols into shift work settings remains challenging. Firstly, the influence of circadian misalignment on cognition is derived from study paradigms that do not simulate night shift work conditions. For instance, in circadian constant routine protocols study participants are kept awake for more than 24 hours while kept in a constant posture under very low light conditions, and circadian forced desynchrony protocols involve recurrent fixed sleep-wake cycles under dim light conditions with cycles that are much shorter or longer than the typical 24-h cycles^14,15^. Furthermore, most of our current understanding of cognitive vulnerability at night builds mostly upon dynamics *within a single* 2*4-h period*^13,16,17^, whereas the effects of circadian misalignment on cognition in shift work are typically experienced *over multiple days*, which limits the translation of these circadian protocols into real-life settings. Secondly, most controlled simulated night work studies have primarily tested cognitive function within a single domain, which is attention resources^18–21^, while in shift work many different cognitive domains are being taxed, dependent on specific components of the work, including cognitive flexibility^22^. Given that task-specific cognitive brain responses show different 24-h dynamics^13^, it is unlikely that all cognitive domains will be (equally) jeopardized over 24-h and with repeated night work. Therefore, one may hypothesize that circadian misalignment, which is typically experienced in night shift workers, impacts cognition task-dependently. Collectively, these gaps of knowledge highlight the need to better understand how the combination of simulated night work (biologically driven by circadian misalignment) and task specificity impact on cognitive performance across multiple work shifts. Thus, the aim of this study was to determine to what extent the misalignment between the endogenous circadian timing system and behavioral/environmental cycles (i.e. wake/sleep, feeding/fasting, light/dark cycles)^23^ - typically experienced by night shift workers - modulates cognition *task-dependently over multiple days*. To address this aim, different cognitive tests were carried out during scheduled wakefulness (Fig. 1) to assess sustained attention, cognitive throughput, information processing, visual-motor performance and declarative memory, which are exquisitely sensitive to increased sleep pressure and circadian phase, and required for optimal performance^12,15,17^.

## Results

**Figure 2:**
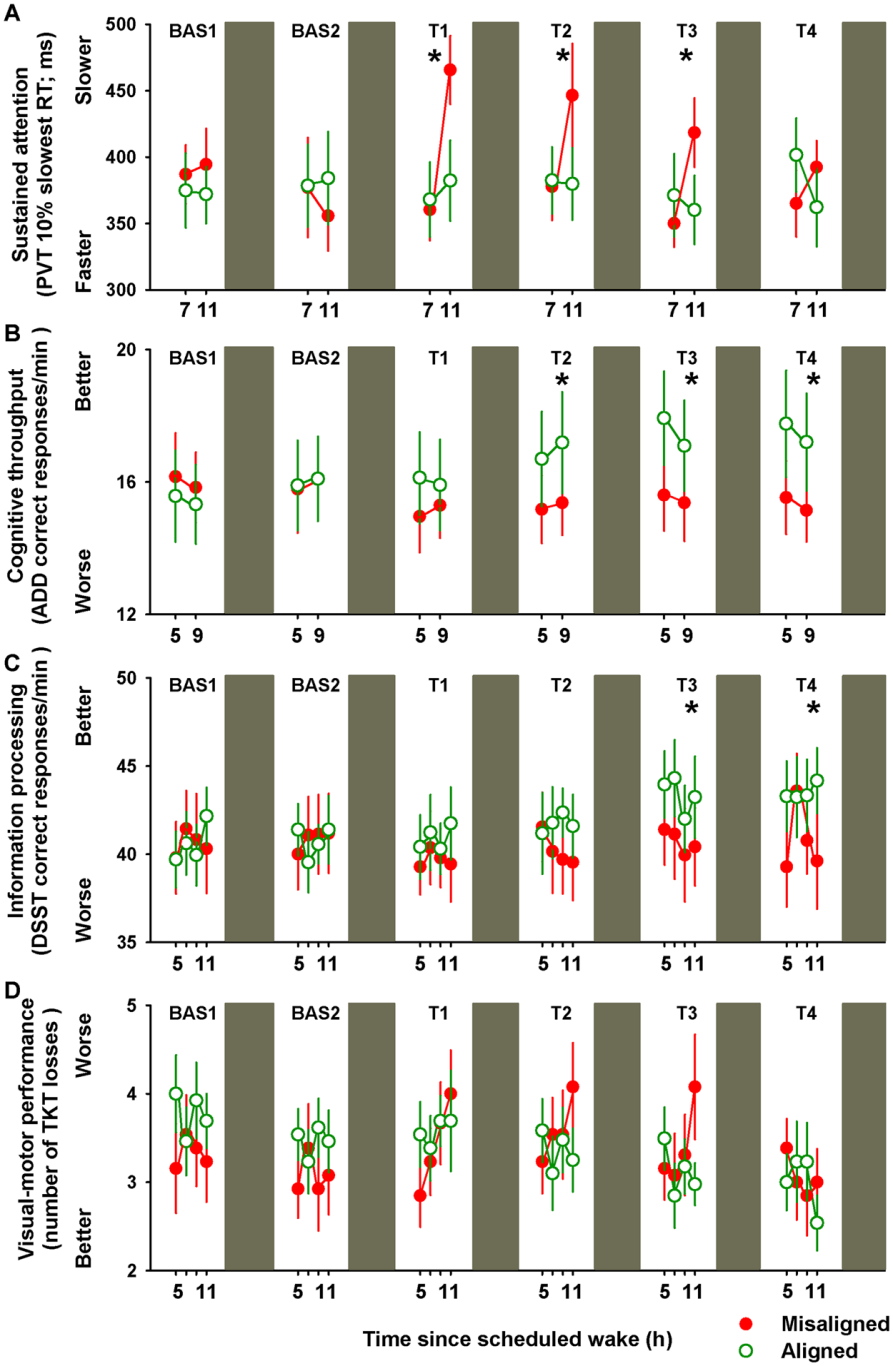
Daily dynamics of cognitive performance under circadian alignment/misalignment. (**A**) Sustained attention (PVT 10% slowest reaction times) worsened following acute circadian misalignment (T1), which lasted up to two days subsequent to it (T2 and T3). See also Figure S1. (**B**) Cognitive throughput (ADD number of correct responses/min) performance improved *only* under circadian alignment for test days 2–4 (T2-T4). (**C**) Information processing (number of correct DSST responses/min) performance improved *only* under circadian alignment during test days 3–4 (T3-T4). (**D**) Visual-motor performance (number of TKT losses) did not significantly differ between circadian alignment/misalignment. Green (open symbols) and red (closed symbols) lines correspond to, respectively, circadian alignment and misalignment conditions. Data correspond to mean ± standard error of the mean (n = 13), *p < 0.05 (see results for statistics).

**Figure 3:**
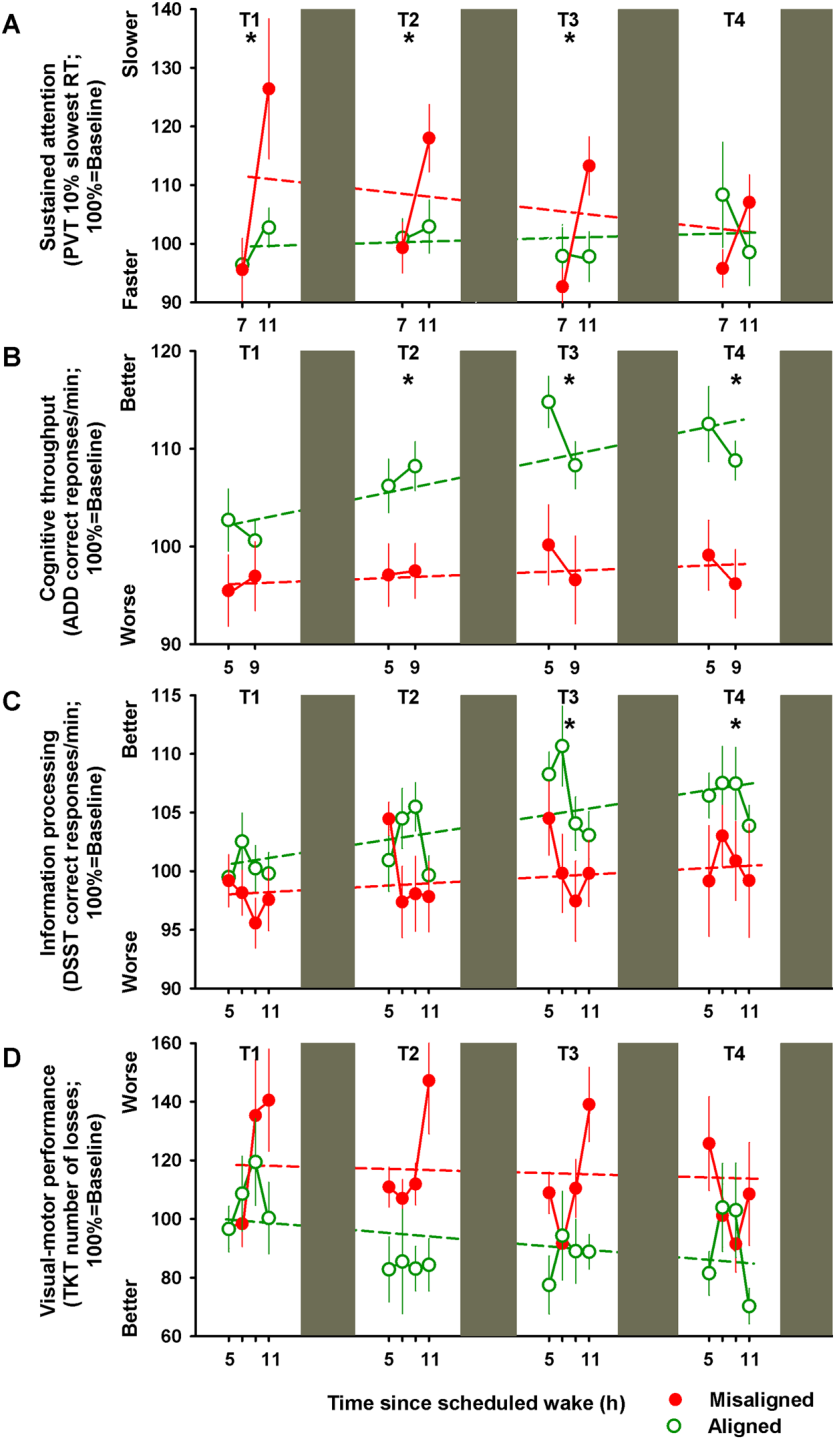
Individual baseline-adjusted cognitive performance across days of circadian alignment/misalignment. (**A**) Baseline-adjusted sustained attention (PVT 10% slowest reaction times) did not change under circadian alignment, while it was slower on the first day of misalignment with subsequent improvement across days. (**B**) Baseline-adjusted cognitive throughput (ADD) performance improved throughout days under circadian alignment, while no daily improvement was observed under misalignment. (**C**) Baseline-adjusted information processing (DSST) performance improved throughout days under circadian alignment, but not under misalignment. (**D**) Baseline-adjusted visual-motor performance (TKT) improved over days under alignment, but not misalignment. Green (open symbols) and red (closed symbols) lines correspond to, respectively, circadian alignment and misalignment conditions. Data correspond to mean ± standard error of the mean (n = 13) (see results for statistics).

We first focused on the effects of circadian misalignment on basic sustained attention, indexed by the Psychomotor Vigilance Task (PVT), as ample evidence speaks to cognitive slowing during the biological night^16,19^. PVT reaction times significantly varied by the interaction of “circadian alignment/misalignment condition”, “test day” and “time since scheduled wake” (F_9,2596_ = 5.5, *p* < 0.001), such that during the first three days (T1-T2), a cognitive slowing was observed under circadian misalignment with median reaction times of ~300 ms when assessed 11 h after scheduled awakening (multiple comparisons post-hoc Bonferroni correction, *p* < 0.05), which was not observed when the same individuals were under circadian alignment (Supplementary Figure 1). The dynamics across multiple work shifts of basic sustained attention highlights the impact of circadian misalignment: PVT performance, as indexed by the 10% slowest reaction times, significantly varied by the interaction of “circadian alignment/misalignment condition” and “test day” (F_5,275_ = 3.3, *p* = 0.006), with the slowest reaction times following acute circadian misalignment (T1), with lasting effects for up to two subsequent days (T2 and T3), and subsequent recovery on the fourth night shift (T4) (Bonferroni correction, *p* < 0.05) (Fig. 2A). To account for individual differences in baseline performance, we normalized each individual’s slowest reaction times to their baseline values. Similar as for the unadjusted analysis, baseline-adjusted sustained attention (PVT 10% slowest reaction times) did not significantly change under circadian alignment (*r*^2^ = 0.05; *p* = 0.6), while it was significantly slower over multiple days of circadian misalignment (*r*^2^ = 0.27; *p* = 0.04) (Fig. 3A).

Cognitive throughput, indexed by the participant’s ability to accurately sum as many pairs of two-digit number as fast as possible^24^ [Addition Task (ADD) number of correct responses/min], significantly varied by the interaction of “circadian alignment/misalignment condition” and “test day” (F_5,276_ = 7.1, *p* < 0.001) (Fig. 2B). Accordingly, cognitive throughput performance improved by ~12% over multiple days of circadian alignment (test days 2–4, as compared to baseline; Bonferroni correction, *p* < 0.05), while no daily improvement occurred when the same individuals were misaligned (*p* > 0.05). In a similar vein, information processing that requires matching symbols and numbers^25^ [number of correct Digit Symbol Substitution Task (DSST) responses/min] significantly varied by the interaction of “circadian alignment/misalignment condition” and “test day” (F_5,558_ = 3.1, *p* = 0.01). We observed a performance improvement of ~12% *only* under circadian alignment during test days 3–4 (T3-T4) (Bonferroni correction, *p* < 0.05) (Fig. 2C). Normalizing to individual baseline performance further confirmed the daily improvement in cognitive throughput and information processing performance *only* during circadian alignment (day shifts) (Fig. 3B,C). Accordingly, baseline-adjusted cognitive throughput [Addition Task (ADD) performance] significantly improved throughout days under circadian alignment (*r*^2^ = 0.61; *p* = 0.02), while no daily improvement was observed under circadian misalignment (*r*^2^ = 0.14; *p* = 0.34). Similarly, baseline-adjusted information processing [Digit Symbol Substitution Task (DSST) performance] significantly improved across days under circadian alignment (*r*^2^ = 0.47; *p* = 0.003), but not under circadian misalignment (*r*^2^ = 0.16; *p* = 0.13).

We subsequently investigated visual-motor performance, indexed by the number of losses participants have during a dynamic-load tracking task, which represents a model of human operational performance, such as in space transportation, whereby complex displays and activities are required^26^. Daily dynamics of visual-motor performance [number of Unstable Tracking Task (TKT) losses] yielded significant differences by the interaction of “circadian alignment/misalignment condition” and “test day” (F_5,560_ = 3.7, *p* = 0.02), although multiple time-series correction indicated no post-hoc significances (*p* > 0.1) (Fig. 2D). However, normalizing to baseline performance indicated significant improvement by ~15% over days under circadian alignment (*r*^2^ = 0.28; *p* = 0.04), which was absent when the same individuals were misaligned (*r*^2^ = 0.04; *p* = 0.43) (Fig. 3D). Lastly, declarative memory, indexed by the capacity to recall six word pairs 10-min after learning^24^ [Probed recall memory Task (PRM) percentage of correct responses], did not significantly vary by the interaction of “circadian alignment/misalignment condition” and “test day” (*p* > 0.1), with similar performance levels over successive days of either circadian alignment or misalignment (Supplementary Figure 2A). Normalizing to individual baseline performance further confirmed that declarative memory performance remained stable over days of circadian alignment and misalignment (respectively, *r*^2^ = 0.03; *p* = 0.69 and *r*^2^ = 0.13; *p* = 0.32) (Supplementary Figure 2B).

**Figure 4:**
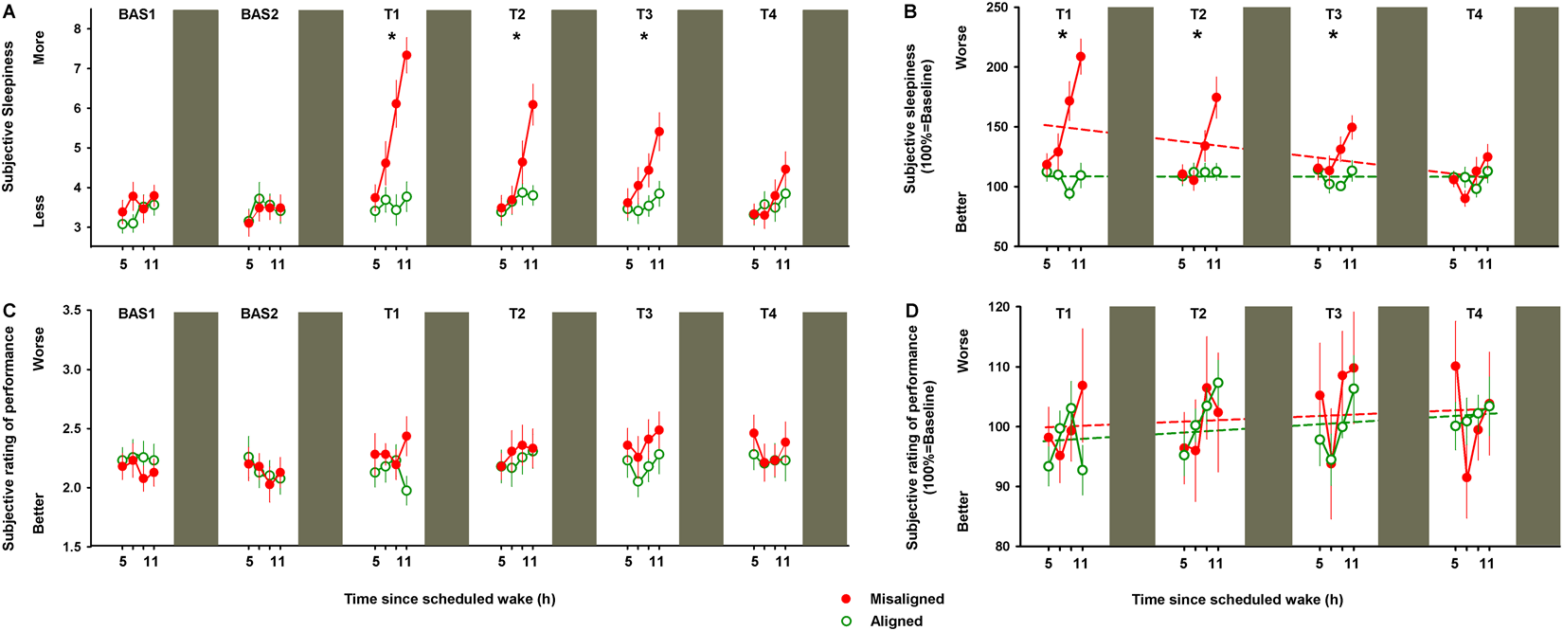
Daily dynamics of subjective ratings of sleepiness and performance under circadian alignment/misalignment. (**A**) Subjective sleepiness (KSS) indicated higher levels of sleepiness during acute circadian misalignment (T1), which lasted up to two subsequent days (T2-T3). (**B**) Baseline-adjusted subjective sleepiness significantly worsened by more than 2-fold on the first day of circadian misalignment with improvement across days, while it did not change under alignment. (**C**) Subjective ratings of performance (PEERS) did not differ between circadian alignment/misalignment. (**D**) Baseline-adjusted subjective ratings of performance did not change under circadian alignment/misalignment. Green (open symbols) and red (closed symbols) lines correspond to, respectively, circadian alignment and misalignment conditions. Data correspond to mean ± standard error of the mean (n = 13) *p < 0.05 (see results for statistics).

**Figure 5:**
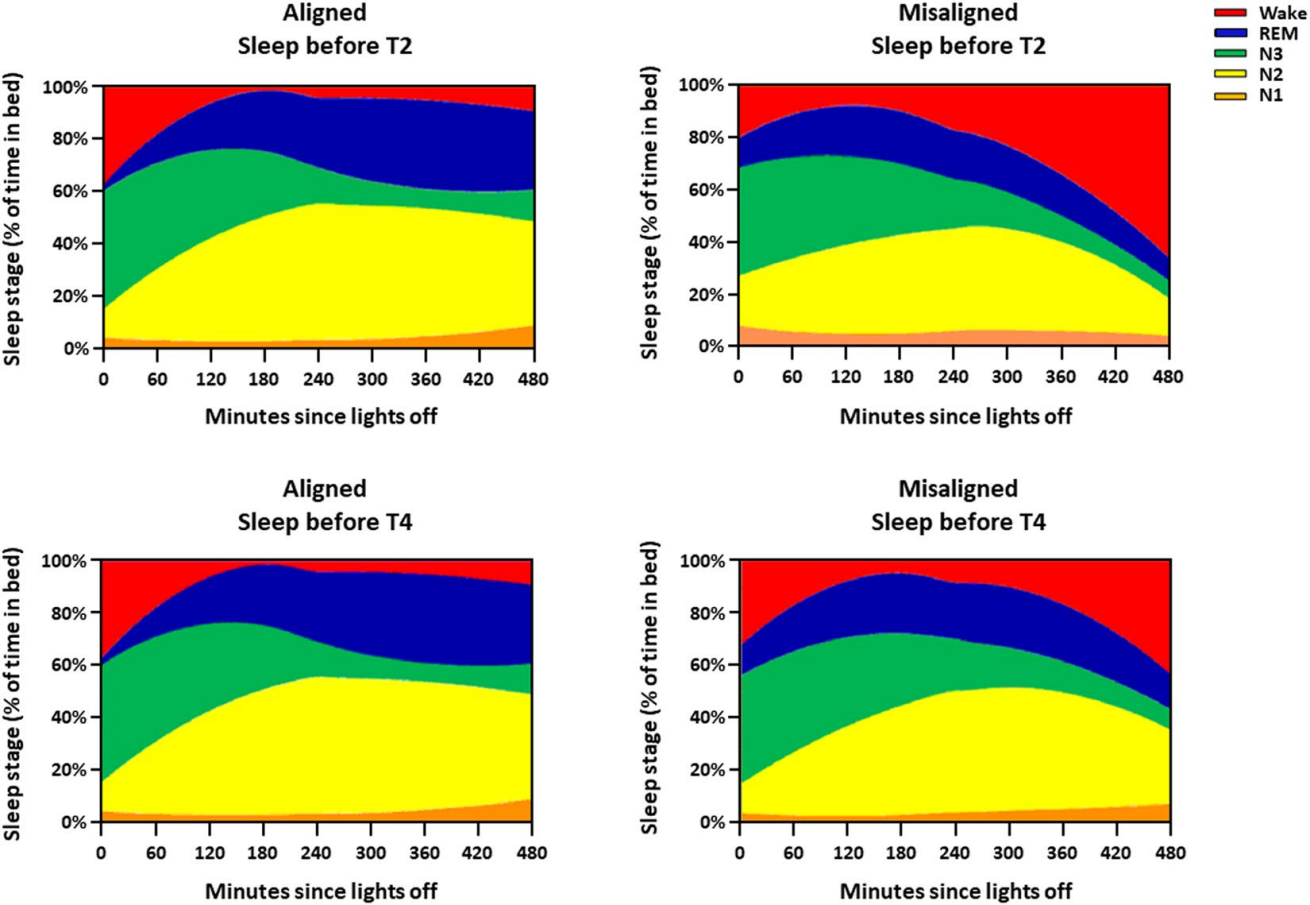
Circadian alignment/misalignment impacts the temporal dynamics of sleep structure. Graphical representation of the local regression analyses that included the interaction of “circadian alignment/misalignment condition”, “time after lights off” and “night”. Stacked areas represent the model-predicted cumulative proportion of each sleep stage (N1-N3, REM and Wake) occurring at each time of the sleep episode under a normally entrained circadian condition (left panels) and under circadian misalignment (right panels) after 1- and 3-days (T2 and T4, respectively) under these circadian conditions. Acute circadian misalignment significantly increased the time-course for the occurrence of wake as compared to circadian alignment (upper panel). After 3-days of misalignment, wake episodes during the last 2-h of scheduled sleep remained higher as compared to when the same individuals were under circadian alignment (bottom panel). Data correspond to mean over all participants (n = 13).

On a next step, we assessed the participant’s subjective ratings of sleepiness and performance. We observed that subjective sleepiness [Karolinska Sleepiness Scale (KSS)] significantly varied by the interaction of “circadian alignment/misalignment condition” and “test day” (F_5,560_ = 12.4, *p* < 0.001), with higher levels of sleepiness during acute circadian misalignment (T1), which lasted up to two subsequent days (T2-T3) (Bonferroni correction, *p* < 0.05) (Fig. 4A). Furthermore, baseline-adjusted subjective sleepiness significantly worsened by more than 2-fold on the first day of circadian misalignment with an improvement across days (*r*^2^ = 0.25; *p* = 0.04), while it did not significantly change under alignment (*r*^2^ = 0.004; *p* = 0.8) (Fig. 4B). Conversely, subjective ratings of performance [Performance evaluation and effort scale (PEERS)] significantly varied by the interaction of “circadian alignment/misalignment condition” and “test day” (F_5,560_ = 3.7, *p* = 0.02), although no significant post-hocs were observed after multiple time-series correction (*p* > 0.1) (Fig. 4C). Baseline-adjusted subjective ratings of performance did not significantly change under circadian alignment (*r*^2^ = 0.12; *p* = 0.18) and misalignment (*r*^2^ = 0.03; *p* = 0.5) (Fig. 4D). On a next step, we investigated whether subjective levels of sleepiness and performance ratings predicted how individuals perform under circadian alignment and misalignment. Subjective sleepiness only predicted decrements in sustained attention (PROC MIXED, analyses of covariance; F_(1,279)_ = 21.9, *p* < 0.001), with no significant predictions on cognitive throughput (PROC MIXED, analyses of covariance; F_(1,271)_ = 3.1, *p* = 0.07), information processing (PROC MIXED, analyses of covariance; F_(1,549)_ = 3.2, *p* = 0.07) and visual-motor performance processing (PROC MIXED, analyses of covariance; F_(1,561)_ = 0.01, *p* = 0.96). Interestingly, subjective ratings of performance did not significantly predict performance vulnerability on sustained attention (PROC MIXED, analyses of covariance; F_(1,185)_ = 0.85, *p* = 0.35), cognitive throughput (PROC MIXED, analyses of covariance; F_(1,177)_ = 1.2, *p* = 0.21), information processing (PROC MIXED, analyses of covariance; F_(1,549)_ = 3.1, *p* = 0.08) and visual-motor performance processing (PROC MIXED, analyses of covariance; F_(1,549)_ = 0.01, *p* = 0.93). Deficits in vigilance and learning under circadian misalignment may be partially due to low circadian drive for sleep during the biological day, with worse daytime sleep^23^ and nocturnal performance^14^. Here we observed that acute circadian misalignment significantly increased the time-course for the occurrence of wake (interaction of “circadian alignment/misalignment condition”, “time after lights off” and “night”; F_14,416_ = 11.4, *p* < 0.001), particularly after 5 h of sleep (Bonferroni correction, *p* < 0.05). After 3-days of misalignment, the percentage of wakefulness during the last 2-h of scheduled sleep remained higher as compared to when the same individuals were under circadian alignment (Bonferroni correction, *p* < 0.05) (Fig. 5). Our circadian misalignment effects on wakefulness during the scheduled sleep episodes speak to a lower sleep ability (i.e., sleep efficiency^27^), particularly during the second half of scheduled sleep. On a next step, we investigated if sleep efficiency impacted on cognitive performance. Lower sleep efficiency in the sleep before cognitive testing significantly impaired sustained attention (PROC MIXED, analyses of covariance; F_(1,78)_ = 4.11, *p* = 0.04), with no similar significant predictions on cognitive throughput (PROC MIXED, analyses of covariance; F_(1,83)_ = 0.04, *p* = 0.84), information processing (PROC MIXED, analyses of covariance; F_(1,83)_ = 0.82, *p* = 0.36), and visual-motor performance (PROC MIXED, analyses of covariance; F_(1,182)_ = 0.18, *p* = 0.66). Therefore, circadian alignment/misalignment effects on some aspects of cognitive performance – sustained attention - depended, at least partially, on prior sleep-wake history.

## Discussion

Here we show, with a biological paradigm mimicking what night shift workers typically experience, that daily circadian misalignment – which was confirmed by each individual’s endogenous melatonin phase assessment^23^ - adversely impacts on cognition, as compared to when the same individuals perform during the day. Importantly, circadian misalignment differentially affected cognitive performance, such that basic sustained attention was acutely impaired and gradually recovered upon repeated exposure to circadian misalignment. Conversely, cognitive throughput, information processing and visual-motor performance during misalignment lost the daily improvements during circadian misalignment that were observed when the same individuals were under circadian alignment. Lastly, declarative memory performance was not substantially affected.

Previous forced desynchrony data indicate an increased rate of deterioration in sustained attention (PVT performance) across wakefulness, particularly during the circadian “night”^24,28^. Similar to those circadian paradigms, our simulated shift work design points to a larger misalignment-induced decline in sustained attention 11 h after scheduled awakening (6AM), as compared to 7 h (2AM). Putative mechanisms include the stronger circadian sleep-promoting signal (6AM vs. 2AM) and homeostatic sleep pressure load (11 h vs. 7 h after scheduled awakening)^16^. Cognitive vulnerability is known to be maximum during the early morning hours^13,29^, and in real-life settings, these sensitive hours correspond to when most operational failures and motor vehicle crashes seem to happen^29^. Neurobiological mechanisms for higher cognitive vulnerability during overnight wakefulness may include increased nocturnal cortical excitability^30^ and a relative increase of GABA_A_ receptor function^31^. Thus, the neuronal milieu during the night may favor sleep by increasing a “GABAergic tone”, potentially leading to cognitive decline. Furthermore, repeated exposure to circadian misalignment was associated with daily dynamic changes in the deployment of basic attention resources, with acute cognitive deterioration and subsequent amelioration over days of misalignment. The dual effects of circadian misalignment and acute sleep loss have been shown to impair sustained and selective attention on the first night of simulated shift work (subsequent to an extended wake period of more than 24 h)^19^. In a similar vein, our data indicate that sleep efficiency significantly predicts sustained attention, such that following the first day of misalignment, lower sleep efficiency predicted slower reaction times in PVT performance. Subsequent recovery in vigilant attention across days of misalignment (see Figs 2A and 3A) may be due to lower homeostatic sleep pressure during the scheduled nocturnal wake period due to improved daytime sleep (see Fig. 5) and/or the circadian system becoming slightly more appropriately aligned with the daytime sleep opportunity^23^. Brain mechanisms accounting for the effects of acute sleep loss (e.g., sleep before test day 2) on sustained attention include adenosine and functional A_1_ adenosine receptor (A_1_AR) availability, which are posited to mediate sleep–wake regulation and cognitive performance^32^. Indeed, recent Positron Emission Tomography (PET) data in humans indicate that extending wakefulness into the circadian night may result in a higher A_1_AR availability in the human brain with concomitant detrimental effects on sustained attention performance^32^.

Our current understanding of cognitive vulnerabilities in shift work paradigms comes from data on vigilant attention^16,18,19,33^. Subjective alertness and objective attention are negatively affected by staying awake at night, as compared to a normal waking day^19,34^. To our knowledge, it is unknown if cognitive abilities including cognitive throughput, information processing, visual-motor performance and declarative memory are hindered by multiple days of simulated shift work. As these cognitive domains heavily depend on the effects of sleep pressure and circadian phase^12,17^, we investigated the impact of circadian misalignment to tasks associated with these cognitive processes. Here we show that, within a single 24-h period, performance on these tasks [including visual-motor task (TKT)] is fairly stable between both circadian conditions, but that across multiple days of circadian misalignment, performance does not progressively improve as observed during circadian alignment. Previous forced desynchrony data indicated increased vulnerability of cognitive throughput (ADD) and information processing (DSST) when individuals perform during the circadian night^24^, and that, across multiple days of circadian misalignment, performance does not improve as compared to when individuals are under circadian alignment^14^. Inherent to the study design of these previous studies and the current study, it is not possible to distinguish whether the impaired improvement across multiple days of misalignment is due to a lack of learning, or that they were unable to express benefits gained from learning. This distinction only becomes visible during recovery. In sleep restriction studies, neurobehavioral deficits (e.g., DSST) induced by consecutive nights of sleep restriction improved with acute recovery sleep, reaching baseline performance levels^35^. Although it may be difficult to rapidly both sleep debt and realign the multi-oscillator circadian system following circadian misalignment, as compared to after ‘simple’ sleep restriction, future studies are needed to test whether the inability to improve cognitive throughput and information processing performance during days of circadian misalignment is restored to baseline levels following sleep recovery and circadian realignment.

Declarative memory performance remained relatively stable across days for both circadian alignment and circadian misalignment, which could be due to a myriad of factors. Any circadian/sleep homeostatic effects on cognitive performance heavily rely on the intrinsic properties/parameters of a given task [e.g., duration of task, speed, timing of when task is administered, “difficulty” (e.g., speed response vs. recall of learned content), and so forth^12^. Furthermore, the impact of circadian/sleep homeostatic effects seems to differ across cognitive domains, with more prominent effects of extended wakefulness into the circadian night on basic sustained attention as compared to working memory performance^14^. These cognitive domain-specific effects may be due to the tasks tapping onto different underlying brain regions, which are themselves differentially affected by circadian misalignment/sleep loss^13^. Indeed, functional magnetic resonance imaging (fMRI-BOLD) cognitive brain function seems to be under local modulation of cerebral circadian phase^13^. These data suggest that the circadian rhythmicity imposed by the master clock, located in the suprachiasmatic nucleus, can be locally altered in cortical and subcortical regions partially due to task-specific requirements, such as task complexity. In other words, not all cognitive brain regions associated to a given task show similar 24-h dynamics. Therefore, one may hypothesize that circadian misalignment impacts cognition task-dependently, as suggested by our differential circadian misalignment effects on basic sustained attention and daily improvement for cognitive throughput, information processing and visual-motor performance.

Potential limitations of our study include the limited number of cognitive tests during each individual’s scheduled wakefulness, as metabolic and cardiovascular measurements were also conducted throughout the study in order to test separate hypotheses^23,36–38^. Given that we only had a limited number of circadian phases and times awake, we may not have captured the combination of highest homeostatic sleep pressure and worst circadian phase. Thus, the circadian misalignment-induced cognitive vulnerability may be even larger than observed. Despite our robust cross-over study design, the relatively low study sample may limit generalizations to a broader population. Lastly, while our study mimicked what shift workers may typically experience daily, it included only 4 days of circadian alignment/misalignment. Thus, the long-term effects of repeated exposure to circadian misalignment on cognitive function remain to be fully established.

Misalignment between behavioral cycles and the internal circadian timing system occurs in many occupational groups in our 24-hr society, including – but not limited to - emergency medical and safety personnel, transportation, and industrial power plants. This misalignment occurs mostly because the internal biological timekeeping system of humans is evolutionary conserved to optimally perform during the day and to support sleep at night. This scenario is at odds with nighttime work^39^, and poses a risk for reduction in productivity and an increased likelihood for accidents^40^. This risk could also be further worsened by a discrepancy between an individuals’ objective performance on tasks versus how they subjectively rate their performance and sleepiness. Attention processes are discussed in light of the interrelationship of subjective perception of alertness and objective attention, such that if an individual experiences impaired objective attention, subjective alertness may or may not also be impaired^18^. For instance, under chronic sleep restriction, individuals who rate themselves as alert and competent can exhibit clear attention deficits^35^. Conversely, observational data using subjective sleepiness scales and measures of vigilant attention indicate that shift workers display substantial sleepiness levels and decrements in vigilant attention while working consecutive 12-h shifts^41^. Here we observed that, while individuals felt progressively sleepier under circadian misalignment albeit rating their performance as unchanged, it did not predict performance impairments across diverse cognitive tasks, similar to that shown during repeated partial sleep restriction^42^. This discrepancy between deterioration of objective performance and a lack of self-awareness in operational settings may pose additional risk for human operational errors. A single exception in our data was that subjective ratings of sleepiness significantly predicted decrements in sustained attention (but no other cognitive function measure), suggesting that participants at least seemed to be aware that they were sleepier when misaligned. Interventions could perhaps target this self-awareness to help workers become attentive to potential impairments.

As nontraditional work schedules beyond the 9AM-5PM timeframe are becoming ever more common, it becomes increasingly important to understand who may be at risk of unintended work-related cognitive impairment, and why. From that knowledge, employers, workers, and practitioners can better craft practical, effective interventions. In this context, our current findings indicate that daily circadian misalignment may help to explain cognitive vulnerabilities experienced by night workers beyond differences in work conditions, and provide a biological framework for the development of countermeasures against adverse cognitive effects in vulnerable shift work populations.

## Methods

Different aspects of this study, which was designed to test separate hypotheses, have been published previously^23,36–38^.

### Participants

Volunteers provided written informed consent and received financial compensation. The study was approved by the Partners Human Research Committee, and was conducted in the Center for Clinical Investigation (CCI) at Brigham and Women’s Hospital (Boston, USA). The study was performed in accordance to the Declaration of Helsinki. Fourteen healthy men and women (20–49 years old) were enrolled (detailed information is provided elsewhere^23,37^). One participant was excluded from the analyses due to low cognitive performance (more than 2 standard deviations from the average group level performance for all cognitive tests) Thus, data presented here include 13 participants (mean age ± SD, 27.8 ± 9.5 y; BMI, 25.5 ± 2.7 kg/m^2^; seven men, six women).

### Study protocol

Participants selected and maintained a normal sleep/wake schedule, with 8-h sleep opportunity for at least 2 consecutive weeks (mean ± SD: 17 ± 3 days) before each laboratory visit. All participants underwent two 8-d in-laboratory protocol, which comprised a randomized, within-participant, cross-over design, where the behavioral and environmental cycle (sleep/wake, fasting/feeding, rest/activity, and dark/light cycles) was either aligned (circadian alignment) or misaligned (circadian misalignment obtained after a rapid 12-h shift of the behavioral cycle) with the endogenous circadian system^23,37^ (Fig. 1). The visits were separated by 2–8 wk (mean ± SD, 4 ± 2 week). On day 1 in each study protocol, participants were admitted at ∼10:30 AM and thereafter remained in an individual laboratory room throughout each laboratory protocol to ensure stringent environmental condition control. During the circadian alignment protocol (Fig. 1, left panel), each participant’s scheduled sleep and wake times occurred between 11:00 PM and 7:00 AM, respectively, throughout the 8-day laboratory setting. During the circadian misalignment protocol (Fig. 1, right panel), each participant’s scheduled sleep and wake times occurred between 11:00 PM and 7:00 AM, respectively, for days 1–3. On day 4, their behavioral cycles were shifted by 12-h, by including an 8-h wake episode and a 4-h sleep opportunity [same sleep opportunity-to-wake ratio (1:2) in both circadian alignment and misalignment protocols]. Thereafter, from day 5 to day 8, each participant’s sleep and wake times occurred between 11:00 AM and 7:00 PM (12-h behavioral cycle inversion), respectively. Light levels during scheduled sleep were set at 0 lx. During the scheduled wake episodes of days 1–3, light levels were ∼450 lx to enhance circadian entrainment; on day 4, light levels were ∼4 lx to assess dim-light melatonin onset; and on days 5–8, light levels were ∼90 lx to simulate typical room light intensity under simulated day and night shift work (circadian alignment and misalignment, respectively) shifts, with the exception of a brief 30-minute 450 lux light exposure to simulate the morning commute, in both circadian alignment and misalignment protocols.

### Cognitive performance and subjective scales

Cognitive testing was distributed in a time window occurring 5–11 h after scheduled wakefulness, thus avoiding potential cognitive impairment driven by sleep inertia (typically up to 4-h after awakening^15,43^). Cognitive performance encompassed sustained attention, cognitive throughput, information processing, visual-motor performance and declarative memory, which are exquisitely sensitive to increased sleep pressure and circadian phase^12,15,17^.

The Psychomotor Vigilance Task (PVT) is a sustained attention performance task sensitive to sleep loss and circadian rhythmicity^16,44^. In the visual PVT, a fixation cross was presented on a black screen. At random intervals (2–10 s), a millisecond counter started, and participants were instructed to press a button to stop the counter as fast as possible. Duration of each PVT lasted 10 minutes. Here we report the slowest 10% PVT reaction times [90^th^ percentile of the RTs between 150 and 500 ms, thus excluding both anticipation errors (<150 ms) and lapses (>500 ms)^45^], as it has been described as a sensitive measure to investigate the effects of circadian misalignment and/or sleep loss on sustained attention^16,45^. The Addition Task (ADD) represents a Calculation Performance task (a measure of cognitive throughput)^24^. Participants are presented with a series of sequential randomly-generated pairs of 2-digit numbers, and are required to sum as many pairs as possible in the allotted five-minute time interval. The Digit Symbol Substitution Task (DSST) is a test of information processing, which assesses cognitive speed and accuracy^25^. The task requires presses on a 9-digit keypad corresponding to a symbol on the computer display screen; instructions include being as fast and as accurate as possible. Duration of task comprised 1.5-min. The unstable tracking task (TKT) is a horizontal axis visual-motor performance task, which indexes operational error^26^. A *target cursor* (crosshair) continuously gravitates to the right or to the left (centered along vertical plane on the display) of the *centerline reference* (broken vertical line; centered along horizontal plane on display) when its position is to the right or to the left of the centerline reference, respectively. We assessed the number of losses (i.e., the number of times that the participant lost control of the cursor) across the three blocks for each session (task duration = 3 min). The probed recall memory (PRM) is a test of declarative memory for unassociated pairs of words, and has been shown to vary with both time awake and circadian phase^24^. The PRM consists of a learning phase during which pairs of unrelated words are presented to the participant to learn, a delay (during which another unrelated, non-word-based task can be given), and a recall phase (after a 10-min delay). Subjective sleepiness was assessed with the Karolinska Sleepiness Scale (KSS)^46^ and subjective rating of performance was indexed by the likert-based Performance evaluation and effort scale (PEERS)^47^, which assessed how participants rated their performance during the cognitive test battery. The order of presentation was fixed across all participants under both circadian conditions.

The cognitive test battery and subjective scales were carried out using the following orders:

- At 5 h and 9 h after time since scheduled wake: KSS-DSST-PEERS-TKT-ADD;
- At 7 h and 11 h after time since scheduled wake: KSS-DSST-PEERS-TKT-PRM (presentation phase)-PVT-PRM (recall phase).

### Polysomnography

Sleep was recorded by polysomnography (Vitaport; TEMEC Instruments), according to the American Academy of Sleep Medicine recommendations^48^—during sleep periods 1, 4, and 6 in the circadian alignment protocol and during sleep periods 1, 5, and 7 in the circadian misalignment protocol. Sleep stages were scored visually per 30-s epochs, according to^48^, by a single experienced polysomnography technician, blind to the circadian alignment/misalignment conditions.

### Data analyses

All statistical analyses were performed with SAS version 9.3 (SAS Institute, Cary, NC, USA). Cognitive data (PVT, DSST, ADD, TKT and PRM) and subjective data (KSS and PEERS) were normalized based on the TRANSREG approach (PROC TRANSREG, SAS). This approach performs transformation regression in which both the outcome and predictor(s) can be transformed, which extends the ordinary general linear model by providing optimal variable transformations that are iteratively derived using the method of alternating least squares. This approach allows for also stabilizing the variance, e.g., the log transform when the SD is proportional to the mean. PROC TRANSREG iterates until convergence, alternating between 1) finding least-squares estimates of the parameters of the model given the current scoring of the data (that is, the current vectors) and 2) finding least-squares estimates of the scoring parameters given the current set of model parameters. This transformation approach was applied to each subjective scale and cognitive test separately, thus considering the linear or non-linear nature (e.g., skewness) of each data set, which would have been substantially overlooked if applying a single transformation type (i.e., log-transformation) across all variables of interest. The relevance of applying this transformational approach is that each cognitive task may vary in their distribution, and using an identical transformational approach may assume that all cognitive tasks have the same statistical nature under a given study paradigm, which may not necessarily be the case. Importantly, appropriate transformation of data prior to the statistical analyses (e.g., mixed-model analyses of variance) significantly minimizes false-positives. Thus, we applied a TRANSREG approach to each cognitive task, which allowed for the detection of the best fit provided by this approach – one of the key outputs of the TRANSREG approach corresponds to a specific lambda coefficient indicating which type of data normalization is required thereafter. Accordingly, the cognitive data were normalized as follows: PVT analyses on reciprocal transformed slowest reaction times, ADD analyses on the ratio of number of correct responses per minute, DSST analyses on the log-transformed ratio of number of correct responses per minute, TKT analyses on the log-transformed number of losses, PRM analyses on log-transformed number of correctly recalled word pairs. For the subjective scale normalization, KSS analyses were performed on the square-root transformed subjective sleepiness data, and PEERS analyses on the raw data of performance ratings. To examine the time-course of cognitive performance and subjective scales, comparisons were made with mixed-model analyses of variance for repeated measures (PROC MIXED, SAS), with main factors “circadian alignment/misalignment condition”, “test day” (BAS1, BAS2, T1, T2, T3 and T4) and “time since scheduled wake” (PVT and PRM: 7-h and 11-h, ADD: 5-h and 9-h, and TKT, DSST, KSS and PEERS: 5-h, 7-h, 9-h and 11-h), and their two and three-way interactions. For the PVT data, we also included a main factor “time-on-task” (first 3 min, second 4 min and last 3 min of the 10-min PVT as for^46^) although no significant effects were observed for this factor and its interaction with the other main factors described above. “Participant” was included as a random factor. Contrasts were assessed with the LSMEANS statement. Because no significant effects were observed for the three-way interaction, but rather for the interaction of “circadian alignment/misalignment condition” and “test day”, we then computed post-hoc multiple comparisons test for this specific interaction, which was adjusted for multiple comparisons using Bonferroni corrections on α ≤ 0.05. Order of circadian alignment/misalignment conditions was included as potential covariate factor, and it did not significantly predict the dynamics of any cognitive task used in this study design. Linear regression models were applied to investigate potential changes across days of circadian alignment/misalignment condition on the dynamics of cognitive performance and self-evaluations of sleepiness and performance (*r*^*2*^; *p*-values with significance set as ≤0.05). We also tested if subject ratings of sleepiness and performance may predict cognitive performance. To that end, covariance analyses (PROC MIXED covtest, SAS) on normalized data were used with factors “subjective rating” (KSS or PEERS), “circadian alignment/misalignment condition”, “test day” and “time since scheduled wake”.

Sleep stage data expressed as percentage of time in bed for subsequent data analyses, using mixed-model analyses of variance with main factors “circadian alignment/misalignment condition”, “night” [night 1 (before T2) and night 2 (before T4], and their two-way interaction. To determine the effects of circadian alignment and misalignment on the probability of occurrence of sleep stages, longitudinal local regressions were applied for each sleep stage (PROC LOESS, SAS). Local regressions allow for the implementation of a nonparametric method for estimating local regression surfaces, by performing iterative reweighting to provide robust fitting. In the LOESS method, weighted least squares are used to fit linear or nonlinear functions of the predictors at the centers of neighborhoods. The radius of each neighborhood is chosen such that the neighborhood contains a specified percentage of the data points. The fraction of the data, called the smoothing parameter, in each local neighborhood controls the smoothness of the estimated surface. Thereof, data points in each local neighborhood are weighted by a smooth decreasing function of their distance from the center of the neighborhood. Subsequently, four separate plots were generated to visualize the regression prediction for the cumulative sleep-stage probabilities during circadian alignment and misalignment during nights 1 and 2, and time-course analyses were performed using mixed-model analyses of variance (PROC MIXED, SAS). Lastly, we tested if the individual ability to sleep (sleep efficiency) predicts cognitive performance under both circadian conditions by using covariance analyses (PROC MIXED covtest, SAS) with factors “sleep efficiency”, “circadian alignment/misalignment condition”, “night” and “time since scheduled wake”.

### Data availability

The datasets generated during and/or analyzed during the current study are available from the corresponding author on reasonable request.

## Electronic supplementary material

Supplementary information
