# Supplementary material for: Daily circadian misalignment impairs human cognitive performance task-dependently
Source: Sci Rep. 2018 Feb 14;8:3041. doi: 10.1038/s41598-018-20707-4 (PMC5812992; doi:10.1038/s41598-018-20707-4)
Supplement: Supplementary file 1 — Supplementary information [file 41598_2018_20707_MOESM1_ESM.pdf]

Daily circadian misalignment impairs human cognitive performance task-dependently

Sarah L. Chellappa[1,2*#], Christopher J. Morris[1,2*], Frank A. J. L. Scheer[1,2#]

[1] Medical Chronobiology Program, Division of Sleep and Circadian Disorders, Departments of Medicine and Neurology, Brigham and Women's Hospital, Boston, MA 02115, USA (institution where work was performed);

[2] Division of Sleep Medicine, Department of Medicine, Harvard Medical School, Boston, MA 02115, USA.

* The authors contributed to this work equally

# Corresponding authors:

Sarah L. Chellappa, Medical Chronobiology Program, Division of Sleep and Circadian Disorders, Brigham and Women's Hospital, Boston, MA 02115, USA; Division of Sleep Medicine, Harvard Medical School, Boston, MA 02115, USA. Email address: schellappa@bwh.harvard.edu

AND

Frank A. J. L. Scheer, Medical Chronobiology Program, Division of Sleep and Circadian Disorders, Brigham and Women's Hospital, Boston, MA 02115, USA; Division of Sleep Medicine, Harvard Medical School, Boston, MA 02115, USA. Email address: fscheer@bwh.harvard.edu

Abbreviated title: Daily circadian misalignment and cognition

Supplementary Figure legends

Supplementary Figure 1. Upper Panel. Under circadian alignment, PVT reaction times did not differ within a day (7h vs. 11h of time since scheduled wake) nor across multiple days, with ~50% of RT observed around 250ms, indicating optimal performance. **Bottom Panel.** Under circadian misalignment, PVT reaction times were slower during the first three days (T1-T2), with ~50% of RT around 300ms after 11h of time since scheduled wake. In all panels, each colored line represents a different day within the study protocol. Data correspond to mean + standard error of the mean (n=13) *$p<0.05$ (see results for statistics).

Supplementary Figure 2. A. Declarative memory performance (PRM percentage of correct responses) did not differ between circadian alignment/misalignment conditions across successive days. **B.** Baseline-adjusted declarative memory (PRM) performance did not differ between days of either circadian alignment or misalignment. Green and red lines correspond to, respectively, circadian alignment and misalignment conditions. Data correspond to mean $\pm$ standard error of the mean (n=13).

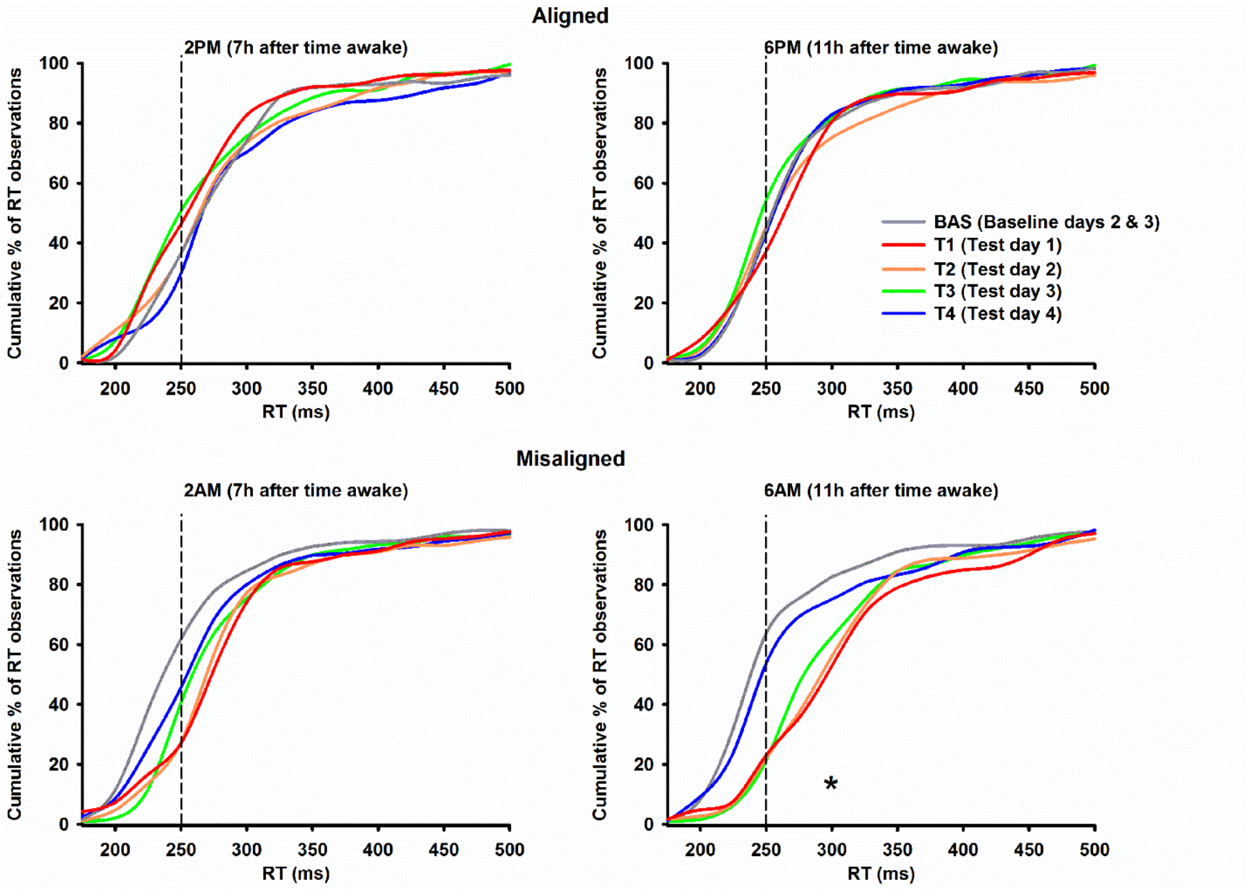

Supplementary Figure 1.

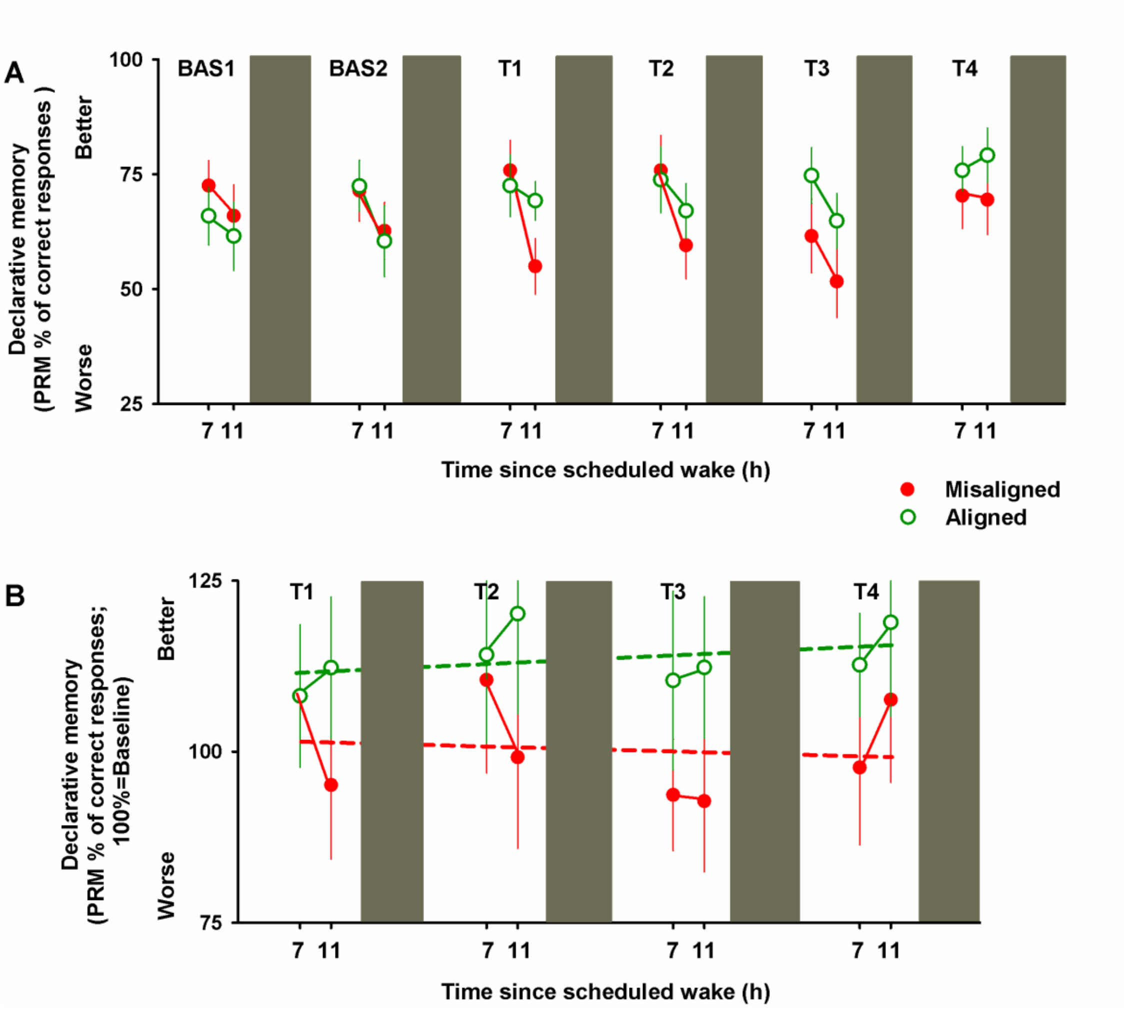

Supplementary Figure 2.
